# Vogt–Koyanagi–Harada Syndrome (VKHS): First Two Cases Reported in Pediatric Age Group in Oman

**DOI:** 10.1155/2023/1745603

**Published:** 2023-10-26

**Authors:** Samiya Al Hashmi, Nasra Al Habsi, Safiya Al Abrawi

**Affiliations:** ^1^Department of Child Health, Pediatric Rheumatology Unit, Royal Hospital, Muscat, Oman; ^2^Department of Ophthalmology, Ophthalmology Unit, Al Nahdah Hospital, Muscat, Oman

## Abstract

The Vogt–Koyanagi–Harada syndrome (VKHS) is a unique form of granulomatous autoimmune disease that mostly impacts the pigmented tissues of the body. The main feature is bilateral granulomatous panuveitis, which is detected on ophthalmologic examination, along with additional systemic signs such as vitiligo, white hair, neurological involvement, or hearing loss. This study aims to report two cases of Vogt–Koyanagi–Harada syndrome presented in the children age group, which is unusual and very rare, to improve recognition of this disease to avoid complications and delay referral.

## 1. Introduction

VKHS is a rare immune condition affecting tissues rich in melanocytes. It is commonly discovered and noted by ophthalmologists during eye examinations. The disease is mainly distinguished by bilateral panuveitis with accompanying neurological and cutaneous manifestations such as vitiligo, alopecia, and poliosis [1–3]. The first VKHS case was reported in 1906 in an adolescent aged 16 who was also suffering from poliosis and iridocyclitis [2, 4]. It is particularly prevalent in populations with darker skin, such as those from Asia, the Middle East, Latin America, and Native Americans, and is uncommon in Caucasians. Most scholars noticed that the condition is more prevalent, at a rate of 2 : 1, among women between the ages of 20 and 50 years [1, 5–7]. Although VKHS affects both children and adults, it is a bit rare among children with aggressive progression of the disease as noticed by Lipps and Khan [7, 8].

Although the risks that lead to the evolution of this disease have not been fully understood, there is evidence to show the involvement of an autoimmune response. Most of the patients are presented in the second and third phases. VKHS diagnosis is carried out through clinical procedures and is best supported by characteristic findings on an ophthalmologic exam [1, 4–6]. The ophthalmologist established the treatment for the patient with an immunosuppressive corticosteroid and was referred to a multidisciplinary team. This included the use of rheumatology to treat acute panuveitis with immunomodulatory and biological medications such as adalimumab, a medication categorized as a “tumor necrosis factor” (TNF) blocker. Caring for a VKHS child can be challenging. The child may experience vision or hearing loss.

Although medication therapy is the primary treatment for the disease, surgical treatment can provide relief from ophthalmologic complications such as subretinal fibrosis, glaucoma, and cataracts. Early identification and treatment can reduce its complications. The prognosis is mostly determined by the phase of the disease at which therapy is undertaken, but it can span from inactive with minimal signs to devastating vision loss. A child may need to see an ophthalmologist, rheumatologist, or dermatologist on a frequent basis. Locally, for the last twenty years of experience in a central hospital, ophthalmologists and pediatric rheumatologists have not seen such a case in the pediatric population. It is undiagnosed and uncommon in Oman. We need to raise awareness in order to establish early treatment and prevent complications.

## 2. Case Description

### 2.1. Case One

**Figure 1:**
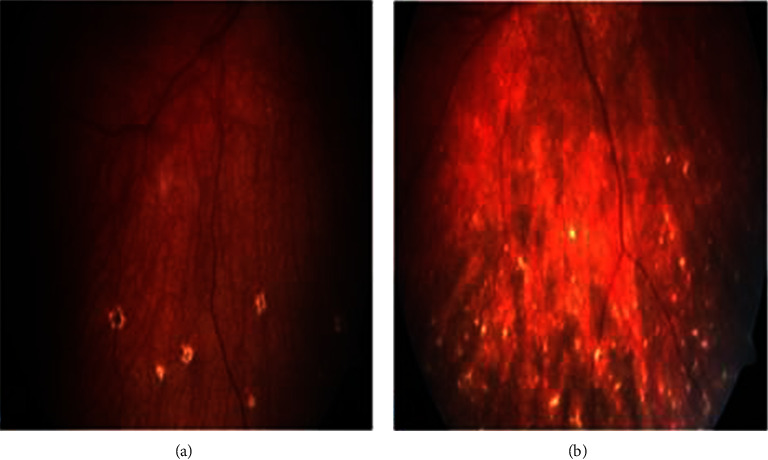
Fundus photograph showing depigmented fundus a sunset-glow fundus (a) and Dalen-Fuchs's nodules (b) in both cases.

**Figure 2:**
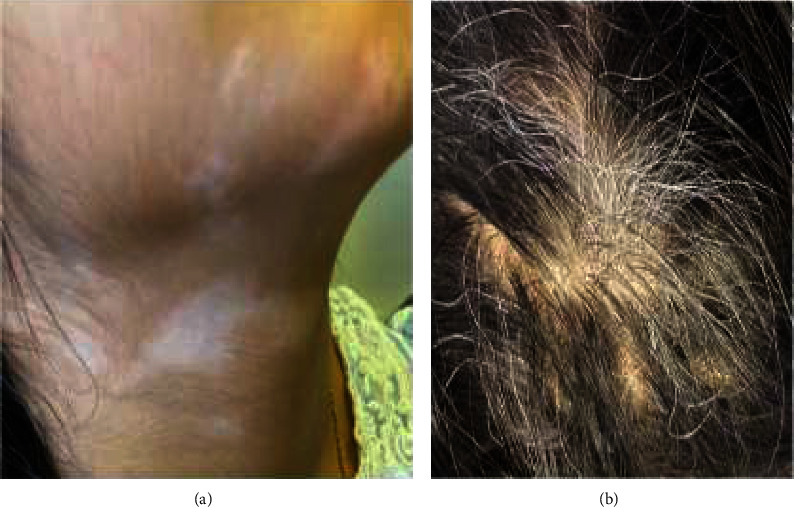
Hypopigmentation of the skin (vitiligo) (a) and gray hair (pilosis) (b) with VKH syndrome (case 1).

A previously healthy nine-year-old girl was diagnosed with Vogt–Koyanagi–Harada disease (VKHS) and panuveitis in April 2021. She presented to local hospitalwith bilateral eye redness, visual blurring, mild headache, low-grade fever, and malaise for two weeks. No other systemic features suggestive of connective tissue disease. Family history of vitiligo in her cousin. General examination was normal. Vital signs noraml. Eye examination revealed visual acuity 6/24 in right eye and 6/9 in the left eye and slit lamp examination revealed bilateral uveitis. Clinical examination was unremakable. Laboratory parameters were normal. Complete blood count and liver and renalfunction tests were within normal range. Autoimmune and infectious disease work up were negative. The diagnosis of bilateral iridocyclitis was made and dexamethasone and tropicamude eye drops were prescribed. Few weeks later she presented with worsening of eye symtoms and eye examination showed bilater granulomatous panuveitis and sunset-glow fundus and Dalen-Fuchs nodules (figure 1). She also developed skin lesion hypopigmented lesion vitiligo, alopecia, and gray hair (pilosis) (figure 2). She was referred to a tertial hospital where a diagnosis of VKHS with panuveitis was made based on ocular exam results and skin findings. She was treated with oral predniskolne 2mg/kg/day plus anti-TNF alfa inhibitor Adalimumab 20mg subcutaneous every 2weeks. (Figure 1(a) (Figure 2). There was a good clinical response and prednisolone was tapered and weaned off. Few months later she had a flare up of eye of eye inflammation while her skin lesion disappeared. Oral prednisolone was restarted and adalimumab 20mg subcutaneous injection was given every week. Last visit was in september 2023 she was seen in out patient clinic. Eye examination revealed active eye inflammation and glaucoma secondary to glucocorticoids.Thre was no alopecia or skin lesion vitiligo or pilosis (gray hair). Adalimumab injection was increased to 40mg once week and oral prednisolne was tapered gradually.Currently she on low dose prednisolone 5mg per day and adalimumab 40mg subcutaneous every week and her disease is well Controlled by anti TNF alfa adalimumab.

### 2.2. Case Two

An eight-year-old Omani girl with a case of Vogt–Koyanagi–Harada disease (VKHS) and bilateral panuveitis was diagnosed by an ophthalmologist in September 2021. Initially, she presented with bilateral red eyes and reduced vision at the ophthalmologic clinic. She denied having any systemic symptoms of autoimmune disease, infectious disease, or rheumatic disease and denied having other ocular or systemic symptoms, such as ocular pain. She denied any family history of a similar presentation or any autoimmune disease. The systemic examination was unremarkable.

The initial eye examination revealed panuveitis, and the fundus showed bilateral disc edema, choroiditis patches, and Dalen-Fuchs's nodules in the midperiphery and periphery with a dry macula (Figure 1(b)). The test results for visual perceptivity were 6/12 in the right eye and 6/18 in the left. She was started on medication by an ophthalmologist with an oral prednisolone dose of 1 mg/kg/day, topical prednisolone 1% eye drops, and atropine sulfate 1% eye drops. Infectious disease work up and autoimmune work were all negative. She was treated with systemic and topical glucocorticoids. She showed improvement in terms of inflammation of the eye.

Three months later, she developed a patch of gray hair (pilosis), alopecia, and vitiligo after tapering the dose of prednisolone. The diagnosis of VKHS with bilateral panuveitis was made based on the constellation of symptoms of panuveitis, gray hair (pilosis), and alopecia and vitiligo (Figure 2). The biological treatment with adalimumab (TNF) blockers was added, and oral prednisolone was restarted. After three months of starting the adalimumab dose of 20 milligrams (mg) every two weeks, there was a dramatic reduction of eye inflammation, and prednisolone was tapered and weaned off.

The frequency of adalimumab injections was spaced every three weeks. She has regular follow-ups with an ophthalmologist and rheumatologist to monitor the disease and the effectiveness and side effects of medication

The last follow-up was in September 2022, and she was clinically doing well with no alopecia, vitiligo, poliosis, or active eye inflammation apart from one eye complication of cataract.

## 3. Discussion and Literature Review

This study involved one of the rare diseases in children and was aimed at raising awareness on early detection and the establishment of early treatment to prevent further systemic complications. VKHS syndrome is an immune condition that affects children and adults of various ages. In this paper, we report the first two cases in the Omani pediatric age group. We described clinical features of this syndrome and the clinical response to anti-TNF alpha inhibitor adalimumab. VKHS is a chronic onset bilateral granulomatous panuveitis condition that is associated with the systemic manifestations of alopecia, vitiligo, pilosis, and central nervous system such as headache and hearing loss [1, 2, 6, 7].

**Figure 3:**
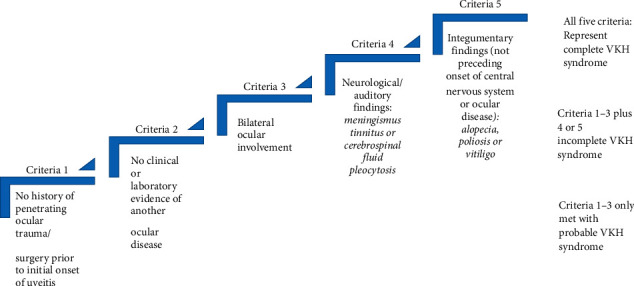
Revised diagnostic criteria of Vogt–Koyanagi–Harada syndrome proposed by the International Nomenclature Committee.

Compared to adults, it is rare in children. The causes of VKHS disease are yet to be discovered. However, several ideas point towards an immune process that is driven by CD4+ T lymphocytes against antigens on melanocytes in the skin, eyes, and meninges as the underlying etiology of VKH illness. Certain members of the major histocompatibility complex type II (MHC II), HLA-DR4, especially HLA-DRB1∗0405, have been found to have a facilitative role in this process [1–3, 7, 9]. Complete VKH, incomplete VKH, and probable VKH are the three key subtypes of the disease. The three subtypes were established after a revision of diagnostic criteria in 2001 by the International Nomenclature Committee [1, 7, 9]. To make a complete diagnosis of VKH disease, symptoms must manifest in all three of the patient's body's major organs: the eyes, the neurological system, and the skin. The presence of neurologic/auditory or integumentary symptoms in addition to ocular involvement characterizes incomplete VKH. In probable type, the eye is the only body part affected (Figure 3) [1, 7, 9].

For a fulfilled VKHS diagnosis, the patient must have no background of ocular trauma as well as no medical or laboratory indication pointing towards the presence of another etiology. In one of our two pediatric cases there was a delay in referral to the multidisciplinary team where the diagnosi of VKHS can be made and appropriate treatment with immunosuppressive medication can be started.. 

The occurrence of VKHS is categorized into four phases. Each of the identified phases is handled through its distinct clinical appearance [1, 2, 4, 7, 10]. Two cases are reported in this study, representing phases two and three of acute and chronic uveitis, respectively. The initial stage is the prodromal stage, characterized by chronic headache, fever, eye discomfort, and photophobia that subsides after a few days. The second stage of panuveitis is known as the acute phase. This stage involves numerous serous retinal detachments (SRD). Stage three chronic develops around three to four months following the commencement of VKH and lasts from weeks to years. Patients typically show hair loss, granulomatous panuveitis, vitiligo, and poliosis of the eyelashes and eyebrows. The fourth stage is the chronic relapsing phase. This stage is characterized by minor ocular and persistent skin and hair lesions [1, 2, 4].

In our case, acute second-stage uveitis alone confused the diagnosis. However, cutaneous signs in both patients later confirmed the diagnosis. Typical ocular findings and skin symptoms are the clinical diagnoses of VKH syndrome. The major complaint presented by our two patients is impaired vision and redness of the eyes. These complaints were followed by skin signs of vitiligo, alopecia, and polio, whereas one patient experienced prodromal symptoms, including headache. In our case, there was no auditory manifestation, such as hearing loss or tinnitus. In contrast, according to a study conducted by AM Abu El-Asrar et al. [5], blurred vision is the primary complaint, along with headache in 14/23 (60.9%) patients, tinnitus in 6/23 (26.1%), and hearing impairment in 3/23 (13%) patients. In addition, some medical researchers have shown that tinnitus and auditory symptoms such as hearing loss account for 50–75% of cases [1, 4, 10].

A clinical diagnosis of VKHS is the gold standard. Conducting further tests on the patient can help rule out other disorders and possibly aid in making a preliminary diagnosis [11]. Although there is no conclusive diagnostic tool for this illness at the present time, tests like a full blood count, erythrocyte sedimentation rates, rheumatoid factor, anti-DNA antibodies, antiphospholipid antibodies, complement, and antinuclear antibody testing can help rule out certain conditions such as rheumatic and inflammatory disease [1, 2, 4, 10]. Since rheumatic disease is a prevalent cause of panuveitis and uveitis, the two cases were thoroughly evaluated for this possibility. However, the results were negative. The disease's progression varies greatly from case to case.

The management of children with VKH syndrome is difficult. Remission rates may be modest, with a significant risk of complications, necessitating the use of long-term immunosuppression. There are presently no established treatment guidelines, and treatment for children with VKH is frequently individualized, despite the fact that several therapeutic modalities have been reported with variable degrees of success [12, 13]. VKHS treatment in children focuses on preventing disease progression by primarily reducing active ocular inflammation that would lead to further complications. The ideal treatment starts with the administration of oral corticosteroids (prednisone at 1-2 mg/kg/day). Thereafter, the patient is slowly tapered to avoid relapse, which is the mainstay treatment of VKHS [1, 2, 7, 12, 14–17]. Children with VKHS who receive appropriate corticosteroid therapy have an exact good prognosis. This treatment may additionally slow the advancement of VKHS, shorten its length, stop serious ocular consequences, and prevent systemic problems which include ear, skin, or hair lesions as noted in current cases.

Secondary immunosuppressants, such as methotrexate, mycophenolate mofetil, azathioprine, or cyclosporine, are considered in patients taking steroids alone or at low doses and can be used to treat ocular disease while steroid doses are reduced [5, 11, 16, 17]. As a result, they hypothesized that methotrexate may be beneficial in producing remission in children with VKH-associated panuveitis with low adverse effects (liver enzyme impairment) [5, 12]. Patients with severe conditions get pulse treatment with methylprednisolone for 3–5 days in total.

Furthermore, patients should take corticosteroids for six to twelve months prior to tapering. It is not recommended to stop using systemic corticosteroids during the first three months due to the risk of relapse [10, 12]. On the other hand, the long duration of steroids may induce eye complications and falter growth. For that reason, a combination of two to three regimens reduced the side effects of the steroid and shortened the duration of steroid treatment without relapsing the condition. Moreover, biological therapies have been shown to treat treatment-resistant uveitis [1, 5, 7, 10, 11, 14, 18, 19]. Adalimumab (ADA) was shown to be a safe treatment, with widespread lymphadenopathy being the only reported side effect. Patients with refractory and severe uveitis benefit greatly from ADA medication, which significantly reduces or eliminates ocular inflammatory activity [11, 13, 17–22]. The medication was proven to be an effective agent in all pediatric patients with nonanterior uveitis over a median follow-up of 17.5 months [11, 13, 21, 22]. In particular, adalimumab (ADA) controlled ocular inflammation in half of the patients and reduced flares in the other half.

Adalimumab (ADA) had a substantial glucocorticoid-sparing impact on the whole group, despite the fact that most of the people in it were rather severely afflicted. ADA may enhance the likelihood of reaching a complete response in cases with posterior uveitis and panuveitis if aggressive therapy is initiated early enough [11]. The benefits of starting adalimumab with steroids in the initial presentation are to prevent relapse and, most importantly, avoid the side effects of prednisone and eye complications [10, 14, 23]. Our cases were presented in the second and third phases of the disease and showed a better clinical response to the biological agent adalimumab (a TNF blocker) along with prednisolone and a dose of 1 mg/kg/day as initial treatment. Furthermore, Jeroudi et al. [14] and Su et al. [23] discovered that patients who received adalimumab and corticosteroids as initial therapy experienced remission of inflammation and a decrease in the need for systemic corticosteroids [11–14, 11, 12, 14, 18, 23–25]. Some complications of VKHS that affect the eyes are cataracts, glaucoma, and chronic serous retinal detachment.

On the other hand, corticosteroid treatment itself can induce side effects in the eye such as posterior subcapsular cataracts, ocular hypertension, and glaucomatous optic neuropathy. First case developed glaucoma secondary to eye inflammation. Therefore, they hypothesized that the combination of methotrexate and adalimumab would be effective in producing remission in children with VKH-associated panuveitis while causing minimal side effects. The disease's progression varies greatly from case to case. Although it looks difficult to recommend immunosuppressive medications as first-line therapy in every case, it may have been advantageous in our two pediatric cases. 

## 4. Conclusion

This paper shows that VKH syndrome is an immune condition that primarily affects the eye as its initial manifestation with etiology is unclear. The eye findings with extraocular manifestations such as vitiligo, peliosis, and alopecia support the diagnosis. Early detection and a multidisciplinary approach can aid in preventing long-term eye problems.

## Data Availability

No data were used to support this study.

## Abbreviations

VKHS: Vogt–Koyanagi–Harada syndrome
VKH: Vogt–Koyanagi–Harada
MHC II: Major histocompatibility complex type II
TNF: Tumor necrosis factor
ADA: Adalimumab
mg/kg/day: Milligram per kilogram per day
mg: Milligrams.
